# Interventional bronchoscopy for benign tracheobronchial diseases under cardiopulmonary bypass support: case reports and literature review

**DOI:** 10.1186/1749-8090-3-27

**Published:** 2008-05-07

**Authors:** Hussamuddin Adwan, Christopher H Wigfield, Stephen Clark, Sion Barnard

**Affiliations:** 1 Department of Anatomy and Developmental Biology, University College London, Gower Street, London WC1E 6BT, UK; 2 Cardiothoracic Unit, Freeman Hospital, High Heaton, Newcastle upon Tyne NE7 7DN, UK

## Abstract

The use of cardiopulmonary bypass as an adjunct to airway surgery for non-malignant diseases in adults is not well established in the UK. We are reporting two cases which demonstrate the additional benefits of using cardiopulmonary bypass during difficult bronchoscopy and complex airway stenting. The first case presents an emergency indication for cardiopulmonary bypass in a life-threatening but benign condition. The second case presented, utilises cardiopulmonary bypass standby as adjunct to a potentially life threatening procedure. A review of the literature is also provided.

## Background

Although the use of cardiopulmonary bypass (CPB) is well established for interventions to treat tracheal stenosis in children, its use during bronchoscopy or tracheal procedures has been less frequently reported in adults. Its use as a standby adjunct to bronchoscopy and tracheal stenting has rarely been reported in the UK.

We would like to report an additional use for CPB for support during bronchoscopy and tracheal stenting for non-malignant diseases.

### Case 1

A 56 year old man presented with progressively worsening dyspnoea at rest. There was no history of chronic lung disease or cyanosis. On examination, he was found to have bilateral chest rhonchi and absent nasal cartilages. Investigation showed reduced FEV1 to 0.8 litres (23% predicted), and within normal basic blood tests. His medications included Prednisolone, Salbutamol and Seretide inhalers, and he was taking Mucolyn syrup regularly. Clinical diagnosis at time of referral was Relapsing Polychondritis, which was further supported by the bronchoscopy findings and a CT scan showing tracheobronchial thickening [1,2]. He underwent endobronchial stenting with a self-expanding metal stent (The Ultraflex™ Tracheobronchial Stent System, Boston Scientific, MA, US) in December 2005 to the trachea and separately to the left main bronchus; post operatively he was well and was discharged after 2 days.

On subsequent review his symptoms showed minimum improvement and his exercise tolerance was approximately 100 yards on the flat. Six months after his first bronchoscopy and stenting he had a silicon stent (The TRACHEOBRONXANE^® ^Dumon^® ^Silicone Stent, Novatech SA, France) inserted into the bronchus intermedius on the right side. During that procedure a subglottic stricture was noted and his airway was found to be very collapsible. Just prior to his planned discharge, he developed noticeable stridor and therefore the stent had to be removed urgently. He deteriorated to type 1 respiratory failure for which he was admitted to the Intensive Care Unit where he was intubated and ventilated. He was extubated and managed with CPAP in High Dependency Unit the following day. Unfortunately, his breathing became more laboured overnight, leading to readmission to Intensive Care Unit and intubation with an un-cuffed paediatric endotrachial tube. A second bronchoscopy was performed and showed significant oedema of the vocal cords, a subglottic stenosis, and the airway stents remained in good position. The distal trachea and right main bronchus were collapsing. There were relatively few secretions, and no evidence of infection. Following his second bronchoscopy and immediately after extubation, he again became hypoxic and witnessed a cardiorespiratory arrest from which he was successfully resuscitated.

He was taken to theatre, as an emergency, and CPB established through right femoral vessels cannulation. Transoesophageal echo was used for control of placement of the long venous cannula. Intra-operative bronchoscopy confirmed collapsing distal trachea. The right and left main bronchi were now seen with copious secretions which were aspirated. A 12 mm × 2 cm uncovered Ultraflex stent was inserted into the bronchus intermedius, and a percutaneous tracheostomy set was used to assist in performing an open tracheostomy. He was weaned from the CPB after 81 minutes, decannulated and the femoral vessels repaired.

He was weaned off the ventilator support over a two week period. Eventually, he required a transfer to a rehabilitation centre. His follow up at six months included a review by an ENT surgeon.

### Case 2

A 53 year old female with a longstanding high laryngo-tracheal post-intubation stenosis underwent elective follow-up bronchoscopy. She was fourteen years post-intubation for multitrauma. In February 2005 she underwent tracheostomy for worsening dyspnoea secondary to tracheo-bronchitis after Staphylococcal sepsis. Her past history was significant for right breast cancer, for which she had mastectomy and chemo-radiotherapy, and pneumothorax as a child.

**Figure 1:**
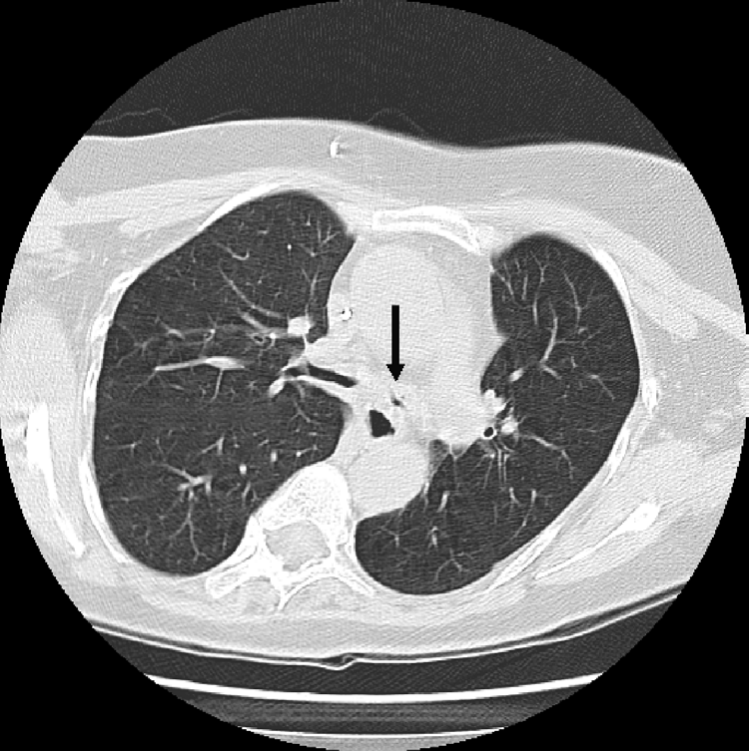
A CT scan section of case 2, showing severe stenosis of left main bronchus.

Bronchoscopy showed severe narrowing of the left main bronchus (Figure 1). Stenting was not attempted at that time, as inflamed mucosa was seen to predispose to bleeding which would have made airway management through the tracheostomy difficult.

**Figure 2:**
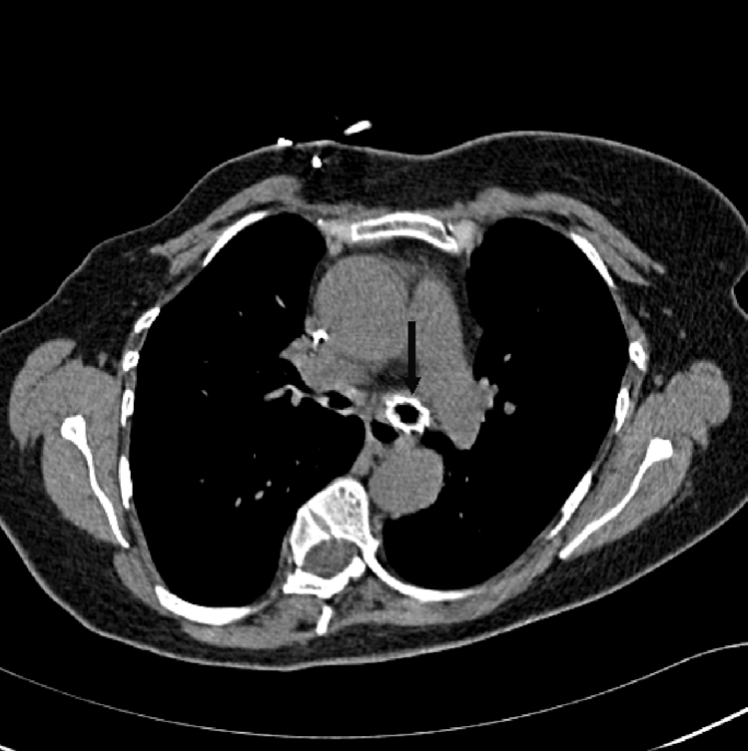
Same patient in Figure 1 after insertion of Ultraflex stent to left main bronchus.

Her preoperative electrocardiogram and her routine bloods including full blood count, urea and electrolytes, creatinin, cardiac enzymes, liver function tests and coagulation profile were within the normal range. She underwent elective bronchoscopy and stenting with Ultraflex, to the left main bronchus with CPB on standby. As a precautionary measure, just prior to airway intervention, the femoral vessels were exposed for potential cannulation. The bronchoscopic procedure was without complications and therefore CPB was not actually required (Figure 2).

## Discussion

Airway stenting has an important palliative role in malignant disease processes. Its usefulness in benign large airway disorders is less well defined. Non-resectable or systemic diseases with benign aetiology causing airway disruption may require high risk bronchoscopy. Airway procedures in this setting carry potential risks, including desaturation, respiratory failure, airway bleeding, stent dislodgement and inability to intubate or establish a definitive airway. Airway manipulation predisposes to other complications, including restenosis, granulation tissue formation, stent migration, metal fatigue, mucus plugging, and halitosis [3,4].

Over half a century, CPB has been refined and used for multiple cardiac and various non-cardiac indications [5]. Since then, CPB equipments have enabled surgeons to perform increasingly complex cardiac and other challenging operations [6]. The use of cardiopulmonary bypass to aid surgery for tracheal procedures is more frequently reported in infants and children than in adult population. There are indications that reports in the literature exploring its use in managing tracheal stenosis in adults are on the increase [7]. The use of CPB is potentially associated with postoperative pulmonary insults, ranging from mild dyspnoea to florid adult respiratory distress syndrome (ARDS) in 1–2% of cases, which in itself carries a 50% mortality rate [8].

To our knowledge there are no clinical reviews in the English literature on the use of CPB in airway management in general, or in airway stenting in particular. Furthermore, most of the published case reports or case series were on the use of femoral-femoral CPB or percutaneous cardiopulmonary support (PCPS) in difficult or compromised airways in tumour-related conditions. These case reports advocate the safe use of femoral-femoral CPB and PCPS in the management of airway obstruction [9-12]. A few case reports from Japan, concerning patients who underwent endotracheal stenting for advanced malignancy utilizing PCPS or Extracorporeal Lung Assist (ECLA) perfusion, have been published [13,14]. The use of PCPS in the palliative surgical management of carcinomatous involvement of the carina to support gas exchange has also been reported from Japan. This allows safe bilateral bronchial dilatation and stenting [15]. Unfortunately, many case reports and case series from Japan are not published in English except for the abstract [16-18].

The cases presented here advocate the safe use of extracorporeal lung support for the treatment of otherwise inoperable tracheal stenoses. Palliative stenting in advanced malignancy is controversial in absence of curative options. In the cases presented here, we found that CPB is safe and useful as a standby during complex stenting procedures for benign tracheo-bronchial conditions with better long term prognosis.

## Conclusion

Utilising support of cardiopulmonary bypass during high risk tracheobronchial interventions for benign diseases is controversial but can be safely applied and can be life saving.

## Competing interests

The authors declare that they have no competing interests.

## Authors' contributions

HA devised the study, acquired and analysed the data, designed and drafted the initial manuscript and prepared the final manuscript for submission to publication.

CW participated in the design, critical revision and writing of the manuscript.

SC and SB helped to conceive the study, provided the data, participated in the design of the study and reviewed and approved the final manuscript.

All authors read and approved the final manuscript.

## Consent

Written informed consent was obtained from the patient for publication of this case report and any accompanying images. A copy of the written consent is available for review by the Editor-in-Chief of this journal.
